# Jack of all trades? Why I chose a training programme in general internal medicine

**DOI:** 10.1016/j.fhj.2025.100466

**Published:** 2025-09-02

**Authors:** Angharad Chilton, Elizabeth Estabrook

**Affiliations:** a ST5 Registrar in General Internal Medicine, University Hospital Southampton, Tremona Road, Southampton SO16 6YD, England; b Consultant in General Internal Medicine, University Hospital Southampton, Tremona Road, Southampton SO16 6YD, England

**Keywords:** General Internal Medicine, Training Programme, Generalist

## Abstract

Within this piece, we discuss the benefits and challenges of being a doctor in the pure general internal (GIM) medicine training programme within the NHS. We argue that having more resident doctors training in GIM within the NHS would provide a wonderful learning opportunity and a sustainable approach to the increasingly multimorbid patient cohort. It is also a discussion of the experience applying for and training within the new GIM curriculum. We discuss some of the future workstreams and consultant opportunities that may be available and compare single CCT GIM with other training courses.

As the Royal College of Physicians is currently encouraging the role of the generalist within medical practice in the NHS, it is hoped that this piece may encourage further discussion on this topic.

Why would someone choose to be in the pilot registrar programme to create a single CCT in general internal medicine for physicians/consultants? I cannot speak about the reasons for others, but the following explains why I don’t regret it one bit.

General internal medicine (GIM) doctors specialise in clinical reasoning and diagnostics alongside the ability to manage complex chronic conditions.^1^ Their patients are often some of the most complex within the hospital due to their physical, mental and social care needs. This means that an ability to balance these concerns is integral to providing excellent standards of care.

Having always been interested in how the body works, I found it difficult to divide human physiology into multiple distinct parts. I never wished to concentrate on one area in isolation because one area simply cannot exist without another. As a result, in my training so far, I have chosen rotations which were general in nature. I found it difficult to understand why the same doctors could not care for patients from their initial presentation to the end of the journey. Why did patients need to see and go over their presenting complaints to physicians working within multiple specialties? I was tempted to specialise in palliative care, but in the end the sticking point was ‘why couldn’t all doctors provide good palliative care?’. General practice was the most varied specialty I could have chosen; however, I knew I wanted to work in a hospital, as part of a team, so that ruled out primary care.

Core medical training was the route I chose as it allowed me to continue to specialise along a general path. Endocrinology initially grabbed me as a career and helped me to move deaneries after personal circumstances meant that a change in training region was essential. I primarily enjoyed it, as it focuses on how hormones and diabetes have effects on all systems. During my rotation, I had an opportunity to work in a consultant-led pure GIM team. This was it! I had found the specialty that gave me what I was looking for. There were complex patients with unknown diagnoses, a team to work with and a space where I could use my communication skills to really aid patient care.

A critic might state: isn’t general medicine already being practised in acute medicine? It most certainly is; however, the expansion of acute medicine over the last few years has meant that they rarely care for patients after the initial diagnosis and stabilisation stage of the patient journey. The Society for Acute Medicine states that they care for patients up to the first 72 h of admission.^2^ They also state that they want to focus on community outreach and same-day emergency care services.^3^ I knew that I wanted to look after patients through their whole hospital journey until they left my care, and work out how I could prevent readmissions.

I also considered medicine for older people. The benefits of geriatric medicine have been well stated – it is recognised that around one-quarter of all bed days in hospital are taken up by patients older than 80 and this is only going to increase with an ageing population.^4^ As more people are becoming multimorbid at a younger age, why should good holistic care be reserved for people who are at a more advanced age? Medicine for older people has tended to look after patients over 80, so I feel there is a gap for patients who do not meet this age cut-off. Having geriatric physicians looking after general medicine patients could pull them away from providing care to patients where frailty was part of their cause for admission.^4^ Those under the age of 80 still represent a substantial portion of hospitalised patients with complex needs. GIM is the career choice that allows me to care for the whole patient, with a broad range of conditions, from beginning to end of their hospitalisation, no matter their age or the complexity of their multimorbidity.

After some time in the GIM department, I was ‘hooked’. However, it was not clear what route I could take to become a consultant. The certificate of eligibility of specialist registration, now known as the portfolio pathway, looked like a feasible option. It would be complicated, but would allow me to become a consultant in general medicine. Fortunately, the establishment of a GIM training programme came at the perfect time and my consultant team really supported me. I applied in the first round and started my training in February 2023. Is it always easy? Of course not. It is lonely at times, and I wish there were more registrar colleagues.

Being part of the general medicine pilot has allowed me to be part of an evaluation trying to understand why people choose this career. I have also been able to witness some of the practicalities involved in starting a training scheme from scratch. The GIM programme evaluation^5^ demonstrated some of the challenges, which included colleagues not knowing that there was now a single accreditation programme or misunderstanding the GIM registrar role. Uncertainty regarding what a GIM consultant job will look like may discourage some from applying.

Several hospital trusts have employed locum consultants to provide GIM care in the recent past, and the development of the GIM training programme has encouraged some to develop dedicated GIM wards and clinical teams, which may solidify the experience of GIM training further. More wards and training programmes have been developed, as shown by the number of training posts available, which has increased from 16 in 2022 (round two) to 36 in 2024 (round one), with 11 available in round two of the same year.^6^

As a specialty develops, it is exciting to consider opportunities for more flexibility to make a varied job plan. This could include roles in management, providing medical consultation to surgical patients,^7^ or in medical education where more educators are needed to instruct medical students and provide holistic care.^8^ Roles focusing on providing clinical care may also be desired, but could this be expanded to be a bridge between primary and secondary care? Several trusts have developed GIM clinics for patients with complex symptoms or for younger patients with multiple conditions causing morbidity. A GIM clinic allows these patients to be assessed as a whole without having to attend multiple healthcare interactions, often without receiving a diagnosis. We know that patients have a more fragmented and stressful clinical journey when they have multiple care boundaries to cross or don’t have continuity with trusted clinicians.^9^ In diabetes and endocrinology especially, the expansion of treatment options still interests me. Might there be a role for general internal medics with a specialist interest, or for trainees to develop specific clinical skills that would provide some variety in a consultant job plan? This process has already begun with the advent of the single-accredited GIM with stroke medicine sub-specialisation.

I do not know fully what my future career will look like, but I do know that every day I go to work I am confident about my passion for providing holistic care to improve patient outcomes. As the programme grows in strength, my hope is that more students and resident doctors will see that GIM is a valid, exciting career and choose to join me in the ranks of physicians training in this specialty. I have heard more often than I would care to count that I am a ‘jack of all trades and master of none!’. Inspiring these future colleagues spurs me onward to ensure that I am a well-rounded and competent physician who can be the master of many!

## CRediT authorship contribution statement

**Angharad Chilton:** Writing – original draft, Investigation, Conceptualization. **Elizabeth Estabrook:** Writing – review & editing, Supervision, Conceptualization.

## Declaration of competing interest

The authors declare that they have no known competing financial interests or personal relationships that could have appeared to influence the work reported in this paper.
